# 93 Foot Burns in Persons with Diabetes outcomes from the National Trauma Data Bank

**DOI:** 10.1093/jbcr/irac012.096

**Published:** 2022-03-23

**Authors:** David Perrault, Jason Cobert, Veda Gadiraju, Geoffrey Gurtner, Tam N Pham, Clifford C Sheckter

**Affiliations:** Stanford University, Palo Alto, California; University of Rochester, Seattle, Washington; University of Washington, Seattle, Washington; Stanford University, Palo Alto, California; University of Washington, Seattle, Washington; Stanford University, Stanford, California

## Abstract

**Introduction:**

Diabetes Mellitus (DM) is an epidemic in the US that complicates the treatment of burn injuries. Lower extremity burns in diabetic patients, particularly the feet, are challenging problems with predictably unfavorable outcomes, as demonstrated by single-institution studies. National evaluations are absent, especially with regard to limb salvage. We aim to characterize lower extremity burns in persons with DM and evaluate the likelihood of amputation. We hypothesize that the incidence of DM associated foot burns is increasing in the US, and these patients are more likely to undergo lower extremity amputation than those without DM.

**Methods:**

The National Trauma Data Bank (NTDB) was queried from 2007-2015 extracting encounters with primary burn injuries of the feet using International Classification of Diseases (ICD) 9^th^ Edition codes. DM is a predefined comorbidity within the NTDB, allowing for cohort comparisons. Logistic regression modeled predictors of lower extremity amputation. Patient covariables included age, sex, race/ethnicity, and comorbidities. Burn covariables included % burn total body surface area (TBSA), mechanism, and region of burn center. Poisson regression evaluated for significance in temporal changes in DM foot burns.

**Results:**

There were 116,796 adult burn encounters of which 7,963 (7%) had foot burns. Of this group, 1,308 (16%) had DM. DM foot burn encounters were older, more likely to be male, and had more comorbidities than non-DM foot burn encounters (all p< 0.001). DM foot burn encounters were more likely to sustain a scald injury (compared to flame) and had smaller %TBSA (all p< 0.001). Additionally, 5.6% of encounters with DM foot burns underwent amputation compared to 1.5% of non-DM encounters (p< 0.001). Independent predictors of lower extremity amputation included DM (OR 3.70, 95% CI 2.98 – 4.59), alcohol use, smoking, chronic kidney disease, burn size >20%, African American/Black race, male sex, and age >40 years (all p< 0.01). The incidence of DM foot burns increased over the study period with an incidence rate ratio (IRR) of 1.09 (95% CI 1.07 – 1.12, p< 0.001).

**Conclusions:**

In the largest cohort study to date, DM was associated with nearly a 4-fold increase in amputation after adjusting for available confounders. Furthermore, the incidence of DM foot burns is increasing. Strategies for optimizing care in persons with DM foot burns are need to improve limb salvage.

